# Flagellum-Mediated Mechanosensing and RflP Control Motility State of Pathogenic Escherichia coli

**DOI:** 10.1128/mBio.02269-19

**Published:** 2020-03-24

**Authors:** Leanid Laganenka, María Esteban López, Remy Colin, Victor Sourjik

**Affiliations:** a Max Planck Institute for Terrestrial Microbiology, Marburg, Germany; b LOEWE Center for Synthetic Microbiology (SYNMIKRO), Marburg, Germany; Yale School of Medicine

**Keywords:** *Escherichia coli*, bacterial physiology, flagellar gene regulation, flagellar motility, mechanosensing, virulence

## Abstract

Bacterial flagellar motility plays an important role in many processes that occur at surfaces or in hydrogels, including adhesion, biofilm formation, and bacterium-host interactions. Consequently, expression of flagellar genes, as well as genes involved in biofilm formation and virulence, can be regulated by the surface contact. In a few bacterial species, flagella themselves are known to serve as mechanosensors, where an increased load on flagella experienced during surface contact or swimming in viscous media controls gene expression. In this study, we show that gene regulation by motility-dependent mechanosensing is common among pathogenic Escherichia coli strains. This regulatory mechanism requires flagellar rotation, and it enables pathogenic E. coli to repress flagellar genes at low loads in liquid culture, while activating motility in porous medium (soft agar) or upon surface contact. It also controls several other cellular functions, including metabolism and signaling. The mechanosensing response in pathogenic E. coli depends on the negative regulator of motility, RflP (YdiV), which inhibits basal expression of flagellar genes in liquid. While no conditional inhibition of flagellar gene expression in liquid and therefore no upregulation in porous medium was observed in the wild-type commensal or laboratory strains of E. coli, mechanosensitive regulation could be recovered by overexpression of RflP in the laboratory strain. We hypothesize that this conditional activation of flagellar genes in pathogenic E. coli reflects adaptation to the dual role played by flagella and motility during infection.

## Abstract (precis)

Flagella and motility are widespread virulence factors among pathogenic bacteria. Motility enhances the initial host colonization, but the flagellum is a major antigen targeted by the host immune system. Here, we demonstrate that pathogenic E. coli strains employ a mechanosensory function of the flagellar motor to activate flagellar expression under high loads, while repressing it in liquid culture. We hypothesize that this mechanism allows pathogenic E. coli to regulate its motility dependent on the stage of infection, activating flagellar expression upon initial contact with the host epithelium, when motility is beneficial, but reducing it within the host to delay the immune response.

## OBSERVATION

Sensing and rapid adaptation to changing environmental conditions are a common survival strategy of bacteria. One of the best-studied examples of a highly regulated cellular function is flagellum-mediated motility. In E. coli, which can exist in various environments and has adopted both commensal and pathogenic lifestyles, the expression of flagellar genes is organized in a hierarchical cascade of three classes that integrate a number of external and internal signals (1, 2). The class I transcriptional regulator FlhDC induces the expression of class II genes, which encode the components of the flagellar hook and basal body as well as a sigma factor, FliA. In turn, FliA is required for the expression of class III genes, which include those for the outer part of flagella and components of the chemotaxis pathway.

Flagella and motility have several functions in bacterial virulence (3, 4). In the early phase of infection, motility is important for moving through the mucus layer and establishing initial contact with the host epithelium, and flagella can serve as adhesins. However, flagella are also highly immunogenic, being rapidly recognized by the innate immune system (5). Known strategies to avoid this immune response used by such pathogens as *Salmonella*, *Campylobacter*, and *Helicobacter* rely on stochastic variation in the expression of flagellar genes, due either to genetic changes (6–10) or to changes at the transcriptional level (11–13). The latter regulation in *Salmonella* is mediated by RflP (formerly called YdiV) (14), which represses *fliA* expression by sequestering the FlhDC complex and targeting it for degradation in a subpopulation of cells (15, 16). Expression of RflP and hence of the fraction on nonmotile cells in the population depends on nutrient levels and cell envelope stress (15, 17).

Although flagellar systems of E. coli and *Salmonella* and their regulation are highly similar, only a few E. coli strains exhibit genetic phase variation in flagellar expression (18, 19). Furthermore, although the *rflP* gene is present in E. coli, it is inactive in laboratory strains because of attenuated translation (20). Thus, it remained unclear what mechanisms might be used by most pathogenic E. coli strains to control motility under conditions that resemble those encountered during infection.

In this work, we investigated the motility of several commensal and pathogenic strains of E. coli grown in liquid or in soft agar. We showed that pathogenic, but not commensal, E. coli strains activate motility only in a porous medium. The underlying regulation requires flagellar rotation as well as expression of RflP, and it is likely to represent a novel mechanism of motility-dependent mechanosensing.

## 

### Pathogenic isolates of E. coli downregulate flagellar gene expression in liquid.

**FIG 1:**
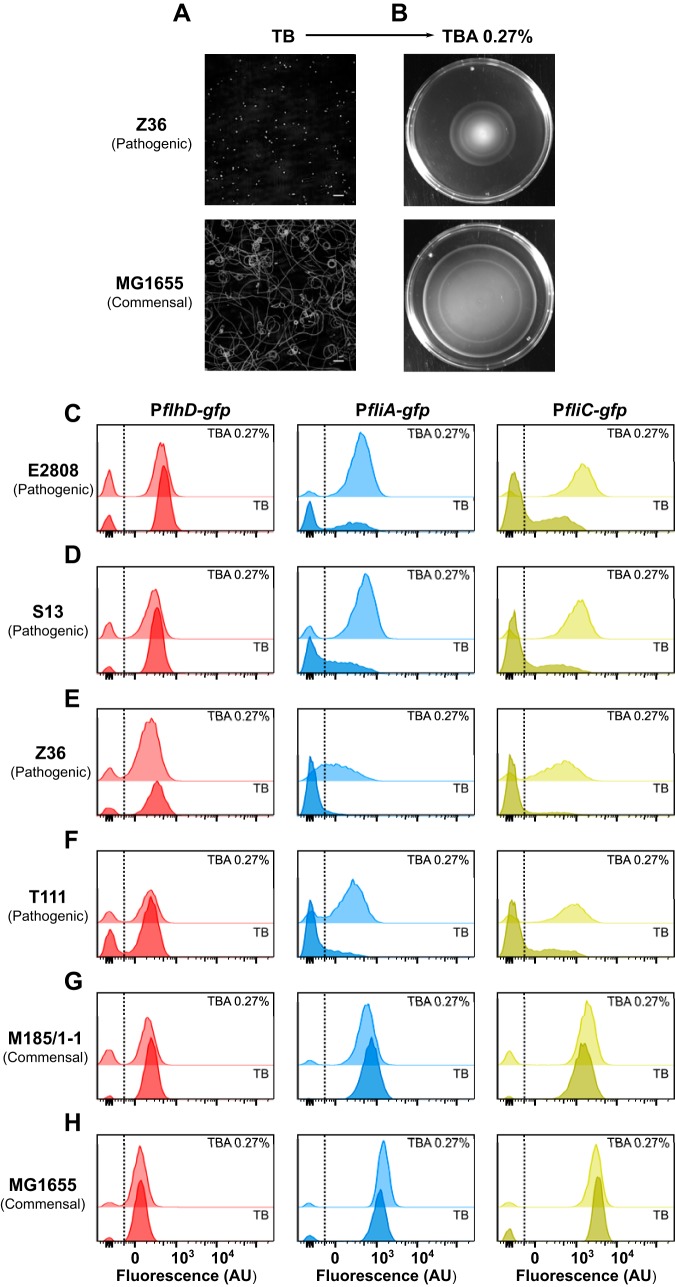
Motilities of pathogenic and commensal E. coli isolates in liquid and in 0.27% agar. (A) Cell trajectories of E. coli Z36 and MG1655, shown as their projected intensities during 20-s-long movies. Bacteria were grown in liquid TB at 37°C with shaking to an OD_600_ of 0.5 to 0.6, diluted to an OD_600_ of 0.05 to 0.1 in TB, and imaged at room temperature. Scale bars, 50 μm. (B) Motility halos formed by E. coli Z36 and MG1655 after 5 μl of the cells grown in TB (as for panel A) was spotted onto the surface of 0.27% TB agar (TBA 0.27%) and incubated at 37°C for 6 to 8 h. (C to H) Distributions of fluorescence levels of P*flhD-gfp*, P*fliA-gfp*, and P*fliC-gfp* reporters in E. coli E2808 (C), S13 (D), Z36 (E), T111 (F), M185/1-1 (G), and MG1655 (H) cells taken from the edges of spreading populations grown in TBA 0.27% or in liquid TB medium, as indicated. Fluorescence was measured using flow cytometry as described previously (46). AU, arbitrary units.

We investigated the motility of a number of pathogenic, as well as a few commensal, natural isolates of E. coli obtained from the Leibniz Institute’s DSMZ collection (Table 1 and see Table S1 in the supplemental material). Surprisingly, while most tested strains (17 out of 19) appeared nonmotile or poorly motile when cultivated in liquid tryptone broth (TB), they became motile on soft (0.27% agar) TB (TBA 0.27%) motility plates, where E. coli cells can spread by swimming through agar pores (Table 1; Fig. 1A and B). Such dependence of motility on growth in agar was confirmed by reinoculation of liquid TB cultures from TBA 0.27% plates and *vice versa*. Interestingly, all E. coli strains following this motility pattern were implicated in pathogenicity, whereas both tested commensal strains from the DSMZ collection and the common laboratory strain MG1655 were motile under both conditions.

**TABLE 1:** E. coli isolates used in this study

E. coli strain^*a*^	Source of isolation or type of pathogenicity^*b*^	Motility on TBA 0.27%	Motility in TB^*c*^
U5/41	UPEC	–	–
DSM 50902	NA	+	–
QST 40139	Possibly commensal strain	–	–
AMC 198	NA	–	–
**E611**	EPEC	+	–
**E2808**	EPEC	+	–
**EW2129-54**	EPEC	+	+/–
**C771**	EPEC	+	–
ICB 4004	Urine	–	–
**S13**	Septicemia patient	+	–
**IHE3035**	Newborn with meningitis	+	+/–
*M185/1-1*	Fecal isolate from healthy individual	+	+
*B185/29-10*	Fecal isolate from healthy individual	+	+
**D699(U5/41 Oac^–^)**	Patient with urinary tract infection	+	–
IHE3043	Newborn with meningitis	–	–
**Z36**	Patient with urinary tract infection	+	–
**T111**	Patient with urinary tract infection	+	–
PC 0886	Possibly commensal strain	–	–
CDC 5624-50	EPEC	–	–
*MG1655*	Commensal lab strain	+	+

a Pathogenic and commensal strains that were motile on TBA 0.27% plates are indicated with boldface and italics, respectively.

b UPEC, uropathogenic E. coli; EPEC, enteropathogenic E. coli; NA, not available.

c +/– indicates that only a fraction of cells are motile.

10.1128/mBio.02269-19.8 TABLE S1 List of bacterial strains and plasmids used in this study. Download Table S1, DOCX file, 0.02 MB. Copyright © 2020 Laganenka et al. 2020 Laganenka et al. This content is distributed under the terms of the Creative Commons Attribution 4.0 International license.

For further analysis of the underlying regulatory mechanism, we transformed four pathogenic and two commensal E. coli strains with plasmids carrying green fluorescent protein (GFP) reporters for all three classes of promoters in a flagellar gene expression cascade. Whereas the activities of the *flhD* (class I) reporters were similar between cells grown in TBA 0.27% and in liquid TB for all strains (Fig. 1C to H and Fig. S1A to F), promoters of *fliA* (class II/class III) and *fliC* (flagellin, class III) were strongly downregulated in liquid TB in pathogenic strains (Fig. 1C to F and Fig. S1A to D). In contrast, no changes in flagellar gene expression were observed in commensal strains (Fig. 1G and H and Fig. S1E and F), consistent with their unchanged motility in liquid culture. Together, these results indicate the existence of a lifestyle-specific motility control in E. coli acting at the level of class II flagellar gene expression.

10.1128/mBio.02269-19.1 FIG S1 Expression of flagellar genes in pathogenic and commensal E. coli isolates in TBA and in liquid TB. Experiments were performed as described for Fig. 1. Percentages of GFP-positive cells in E2808 (A), S13 (B), Z36 (C), T111 (D), M185/1-1 (E), and MG1655 (F) populations are shown. Means from a minimum of four independent replicas are shown; error bars represent standard deviations. *P* values were calculated using the Mann-Whitney test. *, *P < *0.05; ns, not significant. Download FIG S1, EPS file, 0.1 MB. Copyright © 2020 Laganenka et al. 2020 Laganenka et al. This content is distributed under the terms of the Creative Commons Attribution 4.0 International license.

### Rotation of the flagellar motor under a load is required for activation of flagellar gene expression in pathogenic E. coli.

Since the most apparent difference between growth in liquid and that in soft agar is the porous structure of agar, which interferes with flagellar rotation, we hypothesized that an increased mechanical load on the flagellar motor might signal flagellar gene expression in pathogenic E. coli. Such mechanosensing has indeed been described in several bacterial species (21, 22), although its molecular mechanisms remain poorly understood. In E. coli, the load is known to cause posttranslational remodeling of the flagellar motor, increasing the number of force-generating stator units (23), but no gene-regulatory mechanosensing mediated by flagella has been reported so far.

**FIG 2:**
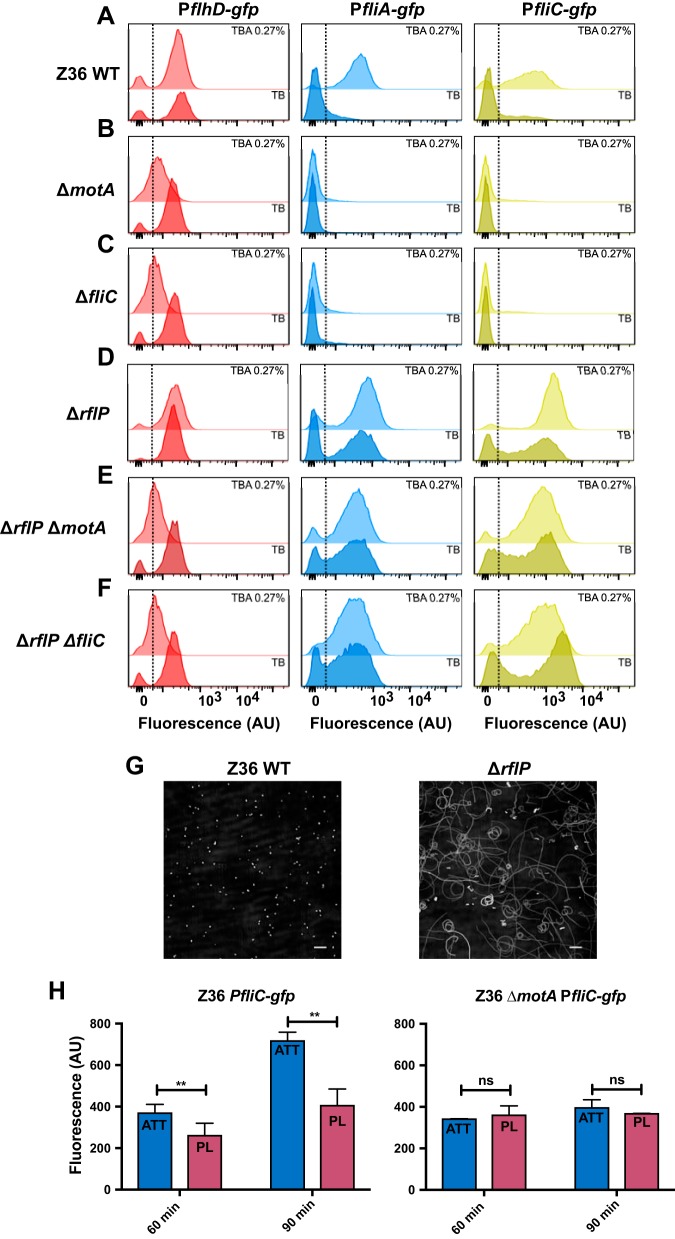
Flagellar motor rotation under a load and RflP control motility in pathogenic E. coli. (A to D) Distributions of fluorescence levels of P*fllhD-gfp*, P*fliA-gfp*, and P*fliC-gfp* reporters in wild-type strain E. coli Z36 (A) and in its Δ*motA* (B), Δ*fliC* (C), Δ*rflP* (D), Δ*rflP* Δ*motA* (E), and Δ*rflP* Δ*fliC* (F) knockout mutants. Bacterial growth and measurements were as described for Fig. 1C to H. (G) Cell trajectories of E. coli Z36 and its Δ*rflP* mutant grown in liquid TB medium, acquired as described in the legend of Fig. 1A. Scale bars, 50 μm. (H) P*fliC-gfp* expression in WT Z36 and Δ*motA* cells forced to the surfaces of microtiter plates (attached [ATT]) by centrifugation or statically incubated (planktonic [PL]), measured by flow cytometry. The mean fluorescence of each GFP-positive subpopulation is shown in arbitrary units (AU). Means from a minimum of four independent replicas are shown; error bars represent standard deviations. *P* values were calculated using the Mann-Whitney test. **, *P < *0.005; ns, not significant.

To test our hypothesis, we made E. coli Z36 knockout strains that lack flagellar filaments and therefore have largely reduced motor loads (Δ*fliC* mutant) or have paralyzed flagella due to the absence of the MotA stator protein (Δ*motA* mutant). These strains were locked in the motility-off state at the level of gene expression in both liquid and agar (Fig. 2A to C; Fig. S2A to C), suggesting that in pathogenic E. coli, rotation of the flagellar motor under a load is indeed perceived as a signal activating the expression of class II and class III flagellar genes. These knockouts could be complemented for both motility and gene expression when respective proteins were produced from a plasmid (Fig. S3).

10.1128/mBio.02269-19.2 FIG S2 Expression of flagellar genes in wild-type and mutant E. coli Z36. (A to D) The percentage of GFP-positive cells in each population and mean levels of fluorescence expressed in arbitrary units (AU) are shown for the wild-type (A), Δ*motA* mutant (B), Δ*fliC* mutant (C), Δ*rflP* mutant (D), Δ*rflP* Δ*motA* mutant (E), and Δ*rflP* Δ*fliC* mutant (F) strains grown and measured as described for Fig. 2. Means from a minimum of four independent replicas are shown; error bars represent standard deviations. *P* values were calculated using the Mann-Whitney test (**, *P < *0.005; *, *P < *0.05; ns, not significant). Download FIG S2, EPS file, 0.2 MB. Copyright © 2020 Laganenka et al. 2020 Laganenka et al. This content is distributed under the terms of the Creative Commons Attribution 4.0 International license.

10.1128/mBio.02269-19.3 FIG S3 Complementation of Δ*motA* and Δ*fliC* deletions. (A) Motility halos formed on TBA 0.27% by wild-type E. coli Z36, the Δ*motA* mutant, and the Δ*fliC* mutant expressing *motAB* and *fliC* under the control of a leaky *trc* promoter without IPTG induction. (B) Distributions of fluorescence levels of P*fllhD-gfp*, P*fliA-gfp*, and P*fliC-gfp* reporters in the wild-type, Δ*motA*, and Δ*fliC* strains expressing *motAB* and *fliC* under the control of a leaky *trc* promoter without IPTG induction. Bacterial growth and measurements were as described for Fig. 1C to H. Download FIG S3, PDF file, 0.1 MB. Copyright © 2020 Laganenka et al. 2020 Laganenka et al. This content is distributed under the terms of the Creative Commons Attribution 4.0 International license.

Besides swimming in porous (such as agar) or viscous media, the mechanical load on the flagellar motor is also expected to increase when bacteria swim toward or attach to a surface. Such surface sensing may indeed be the major function of flagellum-mediated mechanosensing (21, 24). Consistently, we observed that the expression of *fliC* became elevated when wild-type (WT) E. coli Z36 cells were either forced to the surface by centrifugation (Fig. 2H) or grown on the surface of a semisolid (0.5%) agar (Fig. S4). No *fliC* gene induction was observed in Δ*motA* cells under these conditions, suggesting that induction is mediated by the same mechanosensing mechanism. Notably, when cells were grown on a 0.5% agar surface, the *flhD* promoter activity of Δ*motA* cells was the same as that of WT E. coli Z36 cells (Fig. S4). This confirms that *motA* deletion has no direct effect on *flhD* activity and suggests that the lower expression of *flhD* in nonmotile knockout strains grown on TBA 0.27% than in TB (Fig. 2C and D) was rather due to a high cell density and a corresponding nutrient depletion within the nonspreading colony.

10.1128/mBio.02269-19.4 FIG S4 Class I and III flagellar gene expression on TBA 0.5%. Distributions of fluorescence levels of P*flhD-gfp* and P*fliC-gfp* in E. coli Z36 and its Δ*motA* knockout strain grown on TBA 0.5%, quantified as described for Fig. 1C to H. Download FIG S4, EPS file, 0.1 MB. Copyright © 2020 Laganenka et al. 2020 Laganenka et al. This content is distributed under the terms of the Creative Commons Attribution 4.0 International license.

### Anti-FlhC_2_FlhD_4_ factor RflP is involved in mechanosensing.

Since *fliA* and *fliC* are known to be repressed by RflP in *Salmonella*, we tested the effect of *rflP* deletion on the observed regulation. Unlike in the laboratory E. coli strains where RflP had no effect on flagellar gene expression (20), *rflP* deletion in E. coli Z36 resulted in strong derepression of *fliA* and *fliC* expression, particularly apparent in liquid TB medium (Fig. 2D and Fig. S2D). Consequently, most E. coli Z36 Δ*rflP* cells remained motile in liquid (Fig. 2G). This effect could be complemented by expressing RflP from the plasmid (Fig. S5). Deletion of *rflP* also derepressed the flagellar gene expression in the Δ*motA* and Δ*fliC* backgrounds, both in liquid and on TBA 0.27% (Fig. 2E and F and Fig. S2E and F). Although *fliA* and particularly *fliC* expression was still weakly upregulated on soft agar even in E. coli Z36 Δ*rflP*, it remains unclear whether this residual RflP-independent regulation is due to mechanosensing, since to some extent it was also observed in Δ*rflP* Δ*motA* and Δ*rflP* Δ*fliC* double knockouts (Fig. 2E and F and Fig. S2E and F).

10.1128/mBio.02269-19.5 FIG S5 Complementation of *rflP* deletion in E. coli Z36. Distributions of fluorescence levels of P*fliC-gfp* in E. coli Z36 Δ*rflP* expressing an *rflP*-FLAG fusion from an IPTG-inducible promoter, without induction or induced with 10 μM IPTG. Cells were grown in liquid TB as described for Fig. 1. Download FIG S5, EPS file, 0.1 MB. Copyright © 2020 Laganenka et al. 2020 Laganenka et al. This content is distributed under the terms of the Creative Commons Attribution 4.0 International license.

The interplay between RflP-mediated repression and mechanosensing remains to be elucidated (see below). Nevertheless, we observed that the activity of the *rflP* promoter was independent of the cultivation conditions (Fig. S6), suggesting that RflP is not regulated by mechanosensing at the transcriptional level. This is in contrast to the transcriptional regulation of *rflP* by cell envelope stress in *Salmonella* (17).

10.1128/mBio.02269-19.6 FIG S6 Expression of the P*rflP-gfp* promoter in E. coli Z36 in TBA and in liquid TB. Mean levels of fluorescence expressed in arbitrary units (AU) are shown for the wild type and the indicated mutant strains grown and measured as described for Fig. 1. Means from a minimum of four independent replicas are shown; error bars represent standard deviations. *P* values were calculated using the Mann-Whitney test (ns, not significant). Download FIG S6, EPS file, 0.1 MB. Copyright © 2020 Laganenka et al. 2020 Laganenka et al. This content is distributed under the terms of the Creative Commons Attribution 4.0 International license.

**FIG 3:**
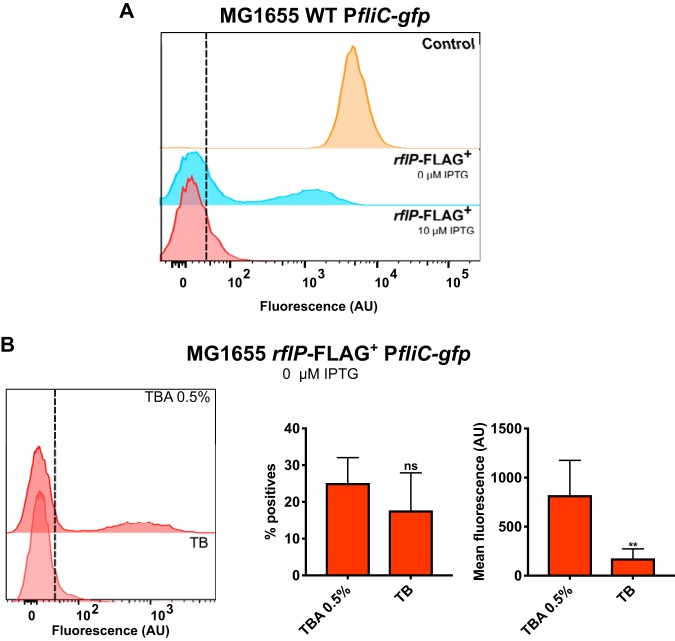
The expression of RflP restores mechanosensing in E. coli MG1655. (A) Distribution of fluorescence levels of P*fliC-gfp* in E. coli MG1655 expressing an *rflP*-FLAG fusion from an isopropyl-β-d-thiogalactopyranoside (IPTG)-inducible promoter, without induction or induced with 10 μM IPTG. Cells were grown in liquid TB as described for Fig. 1. (B) Distribution of fluorescence levels of P*fliC-gfp* in MG1655 cells expressing RflP, grown on TBA 0.5% or in liquid TB. Media were supplemented with ampicillin to prevent plasmid loss. Fluorescence was quantified as described for Fig. 1. Means from a minimum of seven independent replicas are shown; error bars represent standard deviations. *P* values were calculated using the Mann-Whitney test (**, *P < *0.005; ns, not significant).

Importantly, our results also indicate that the determinants of mechanosensing other than RflP are present in E. coli MG1655. The overexpression of *rflP* not only led to the expected dose-dependent inhibition of *fliC* expression in MG1655 (Fig. 3A) but also apparently restored mechanosensing in this laboratory strain, with elevated *fliC* expression in soft agar compared to that in liquid (Fig. 3B). Since the inhibition of *fliC* expression by RflP was only partial in MG1655, this increase was particularly pronounced at the level of mean reporter activity rather than as a fraction of positive cells.

### Proteome regulation by mechanosensing.

In order to identify nonflagellar proteins that could be regulated by mechanosensing, we performed comparative whole-proteome analysis of WT E. coli Z36 and Δ*motA* cells grown either on 0.5% agar or in liquid TB medium. Apart from flagellar motor proteins, several metabolic enzymes and uncharacterized proteins were upregulated in a *motA*-dependent fashion on the agar surface, whereas several transporters and metabolic enzymes, as well as the sensor kinase QseC, were downregulated (Table 2). Since RflP could not be detected in these samples, its regulation at the protein level could not be assessed. Moreover, the expression of many other proteins, some of them involved in the acid stress response, was upregulated on the surface in a *motA*-independent manner (Table S2). Since QseC, a member of the QseB QseC two-component regulatory system, was among the most strongly downregulated proteins and it was previously shown to play a role in the regulation of flagellar expression (25, 26), we tested whether this system might have a regulatory role in mechanosensing. However, no effect of *qseC* deletion on *fliC* expression could be observed in E. coli Z36, grown either on soft agar or in TB (Fig. S7), suggesting that under our conditions and in this strain, this two-component system is inactive. Thus, QseC does not seem to have a mechanosensory role. Nevertheless, its mechanosensitive regulation by pathogenic E. coli might be important during infection, since QseC was shown to be important for virulence (26, 27).

**TABLE 2:** Proteins regulated by motility-dependent surface sensing in E. coli Z36^*a*^

Protein	Function	Fold change between:		
		Δ*motA*_surf_ and WT_surf_	WT_liq_ and WT_surf_	Δ*motA*_liq_ and Δ*motA*_surf_
YhdP	Uncharacterized protein	–21.18	–177.91	–1.13
IroE	Uncharacterized protein	–5.49	–78.78	1.60
PrpC	Citrate synthase	–31.93	–24.14	1.13
PrpD	2-Methylcitrate dehydratase	–4.96	–5.62	–1.03
PrpE	Propionate-CoA ligase	–3.92	–5.34	–1.21
FliH	Flagellar assembly protein	–3.71	–2.22	1.77
FliS	Flagellar protein	–3.20	–2.10	1.88
ElaB	Uncharacterized protein	7.66	–1.61	–2.15
MotB	Flagellar stator component	–5.02	–1.27	2.75
UbiB	Probable protein kinase	29.56	1.00	–1.25
YfiQ	Uncharacterized protein	7.61	2.32	–1.52
UidR	HTH-type transcriptional regulator	3.65	2.51	–1.63
C2460	Putative polyketide synthase	3.56	3.42	1.64
TdcF	Putative reactive intermediate deaminase	3.34	3.54	1.09
AnmK	Anhydro-*N*-acetylmuramic acid kinase	3.10	3.75	–1.15
YffB	ArsC family protein	4.44	4.07	1.81
Gst	Glutathione *S*-transferase	4.05	4.78	1.19
Alr	Alanine racemase, biosynthetic	4.60	5.11	1.19
ThiQ	Thiamine import ATP-binding protein	4.81	5.46	1.10
NrdG	Anaerobic ribonucleoside-triphosphate reductase-activating protein	5.57	6.79	1.19
SdhE	FAD assembly factor	5.20	7.30	–1.88
MobB	Molybdopterin-guanine dinucleotide biosynthesis protein	4.95	7.76	2.42
C3307	Putative conserved protein	5.65	10.05	1.15
KefC	Glutathione-regulated potassium efflux system protein	22.47	10.50	–1.53
YheV	Uncharacterized protein	4.34	11.64	1.94
C1760	Putative transcriptional repressor	19.74	13.14	–1.12
SapA	Peptide transport periplasmic protein	19.54	18.54	1.46
NusB	N utilization substance protein	17.48	20.27	1.60
YddE	Uncharacterized protein	29.57	21.36	–1.88
C4017	Putative ribose ABC transporter	6.30	22.17	2.92
YjjU	Uncharacterized protein	77.64	37.40	–1.68
YbbL	Hypothetical ABC transporter ATP-binding protein	26.50	43.96	2.04
YcjZ	Hypothetical transcriptional regulator	5.53	63.19	1.70
IdnK	Gluconokinase	33.19	84.34	2.27
ThiJ	4-Methyl-5(b-hydroxyethyl)-thiazole monophosphate biosynthesis enzyme	163.82	111.05	–1.09
CyoC	Cytochrome *bo_3_* ubiquinol oxidase subunit 3	69.54	130.08	1.47
YgiY	Uncharacterized protein	103.01	132.15	1.01
QseC	Sensor protein	103.01	132.15	1.01
RlmC	23S rRNA [uracil^747^-C^5^]methyltransferase	92.80	166.17	2.17

a Proteins were selected from the whole-proteome data based on the following criteria: differential expression between the WT and the Δ*motA* mutant (fold change, ≥3) on TBA 0.5%, differential expression between the WT on TBA 0.5% (WT_surf_) and in liquid TB (WT_liq_), and little or no difference (fold change, <3) between the Δ*motA* mutant on TBA 0.5% (Δ*motA*_surf_) and in liquid TB (Δ*motA*_liq_). The E. coli CFT073 protein annotation database was used as a reference. CoA, coenzyme A; HTH, helix turn helix; FAD, flavin adenine dinucleotide.

10.1128/mBio.02269-19.7 FIG S7 Sensor kinase QseC is not involved in mechanosensing. (A) Distributions of fluorescence levels of P*fliC-gfp* in E. coli Z36 and its *qseC*::Km knockout strain grown in TBA 0.27% or in TB, measured as described for Fig. 1. (B) Corresponding percentage of GFP-positive cells in each population. Means from a minimum of four independent replicas are shown; error bars represent standard deviations. *P* values were calculated using the Mann-Whitney test (***, *P < *0.0005; **, *P < *0.005; *, *P < *0.05; ns, not significant). Download FIG S7, EPS file, 0.1 MB. Copyright © 2020 Laganenka et al. 2020 Laganenka et al. This content is distributed under the terms of the Creative Commons Attribution 4.0 International license.

10.1128/mBio.02269-19.9 TABLE S2 Proteins with MotA-independent changes in expression between growth on TBA 0.5% and that in liquid TB as revealed by whole-proteome analysis of E. coli Z36. Shown are only proteins with a ≥3-fold change between samples. Download Table S2, DOCX file, 0.02 MB. Copyright © 2020 Laganenka et al. 2020 Laganenka et al. This content is distributed under the terms of the Creative Commons Attribution 4.0 International license.

In this study, we demonstrate that the expression of flagellar genes in a number of pathogenic E. coli strains is inhibited in liquid but that their expression is induced when bacteria are grown either in porous medium or on a surface. Because this regulation was not observed in commensal or laboratory strains of E. coli, it likely represents a specific mechanism to avoid the adaptive immune response of the host, akin to genetic or transcriptional variation in the expression of flagellar genes observed in other bacterial pathogens (6–12, 28). However, unlike with the stochastic antigenic variation or transcriptional switching observed in these bacteria, the control of flagellar gene expression in pathogenic E. coli apparently relies on sensing the mechanical load on the flagellar motor.

Although surface sensing-mediated control of gene expression, including control of bacterial flagellation, was reported before, most notably in *Vibrio*, *Caulobacter*, and *Bacillus* species (24, 29–35), this is, to our knowledge, the first example of such regulation in E. coli. It was previously shown that enterohemorrhagic E. coli (EHEC) can regulate the expression of EHEC-specific virulence genes upon sensing host attachment and fluid shear (36), but this surface sensing is not known to rely on flagella or to regulate motility. Moreover, this gene-regulatory mechanosensing controls motility in a different way than was previously described for posttranslational remodeling of the E. coli flagellar motor under a mechanical load (23, 37).

We hypothesize that such motility-dependent mechanosensing allows pathogenic E. coli to express flagella upon contact with epithelial cells or in mucus when motility is beneficial for the initial attachment to the host but that pathogenic E. coli shuts down the expression of flagellar genes upon entry into the host in order to delay detection by the immune system. In line with apparent trade-offs of flagellin expression in pathogenic E. coli, we also observed bimodal flagellar expression in these strains, with a subpopulation of cells remaining in the flagellum-off state even in soft agar. A similar bimodal expression was previously observed in other bacteria, including *Salmonella* (16) and Bacillus subtilis (38), but whereas in *Salmonella* bimodal *fliC* expression is known to depend on RflP activity (16, 17), deletion of *rflP* in E. coli Z36 did not abolish the bimodality of *fliC* expression, suggesting a different origin of bimodality.

Though the underlying mechanism of signal transduction from the flagellar motor to transcriptional regulation remains to be established, it apparently requires flagellar rotation under a load. The motility-off state of gene expression was observed not only for the filament-deficient Δ*fliC* strain of E. coli that cannot efficiently sense the load but also for the Δ*motA* strain, which has immobilized flagella. This contrasts with observations of B. subtilis and Vibrio parahaemolyticus; these organisms with deletions of motor proteins mimic strains under high loads (30, 32), but their phenotype is similar to the lock-off mechanosensing phenotype of *mot* mutants of Caulobacter crescentus and Pseudomonas aeruginosa (24, 39).

Our results demonstrate that mechanosensing occurs in pathogenic E. coli strains due to the activity of the motility repressor RflP, which in these strains inhibits the expression of flagellar genes in liquid. Consistently, artificial upregulation of RflP in E. coli K-12 unmasks its mechanosensing response, demonstrating that other sensory determinants are present in the laboratory strain. While at this stage it cannot be ruled out that RflP-stimulated proteolysis of the FlhDC complex is required only to generally lower the basal expression of flagellar genes, its involvement in mechanosensing might also be direct, as occurred with a previously reported surface contact-dependent relief of proteolysis of a master flagellar regulator in B. subtilis (31).

### Bacterial strains and culture conditions.

All strains and plasmids used in this study are listed in Table S1. E. coli strains were grown in liquid tryptone broth (TB) medium (10 g tryptone and 5 g NaCl per liter) or in lysogeny broth (LB) medium (10 g tryptone, 10 g NaCl, and 5 g yeast extract per liter) supplemented with antibiotics where necessary.

### Cloning and knockout strain construction.

In order to construct the *rflP-gfp* fluorescent reporter, the bp –253-to-bp +90 promoter region of the *rflP* gene was amplified from the E. coli Z36 chromosome and cloned into the pUA66 vector using XhoI and BamHI restriction sites (40). For *rflP*-FLAG plasmid construction, the *rflP* gene sequence without a stop codon was amplified from the E. coli Z36 chromosome with a FLAG coding sequence, with the stop codon being added via a primer overhang. The resulting fusion construct was cloned into the pTrc99A vector using SacI and XbaI restriction sites (41).

All the knockout strains described in this work were obtained using the lambda red recombination system as described previously (42). Where necessary, kanamycin cassettes were flipped out using FLP-FLP recombination target (FRT) recombination (43).

### Motility assay in soft agar.

Single colonies of different E. coli strains were transferred onto the surfaces of soft agar plates (TB containing 0.27% agar), and the motile behavior of E. coli strains was assessed after 6 to 8 h of incubation at 37°C by analyzing the motility halo diameter. The cells from the edges of such halos were subsequently transferred into 3 ml of fresh TB medium and grown with shaking at 37°C to an optical density at 600 nm (OD_600_) of 0.5 to 0.6. The motility of the cells was then analyzed using phase-contrast microscopy.

### Single-bacterium movement tracking.

E. coli MG1655, Z36, and Z36 Δ*rflP* overnight cultures were diluted in fresh TB and grown at 37°C with shaking to the OD_600_ of 0.6. The samples were subsequently diluted to final OD_600_s of 0.05 to 0.1 in TB and loaded into ibidi channels (μ-Slide Chemotaxis^3D^; ibidi GmbH, Germany). Movies were recorded at ×10 magnification with a phase-contrast microscope at a rate of 20 frames per second using a complementary metal-oxide semiconductor (CMOS) camera (exposure time, 1 ms), normalized by dividing each frame by an image of the background illumination under the same conditions and inverted in order for the cells to appear bright. A projection of the temporal maximum of each pixel was computed to produce an image of the cell trajectories.

### Flow cytometry.

Promoter activities of the flagellar genes *flhD*, *fliA*, and *fliC* and the motility regulator *rflP* were assayed using plasmid-based reporters containing the respective promoter regions fused to *gfp* (Table S1). Promoter activities were analyzed in the cells collected from the edges of motility halos formed on 0.27% TBA plates and after subsequent cultivation in liquid TB medium. The samples were diluted in 2 ml tethering buffer (10 mM KH_2_PO_4_, 100 μM EDTA, 1 μM l-methionine, and 10 mM sodium lactate, pH 7.0), and fluorescence was measured with a BD LSRFortessa SORP cell analyzer (BD Biosciences, Germany).

In order to compare P*fliC-gfp* activity in attached and planktonic cells, 300 μl of WT Z36 and Δ*motA* mutant day cultures in TB (OD_600_ = 0.6, diluted in fresh TB to the final OD_600_ of 0.05) were inoculated into the wells of a 96-well microtiter plate (ibidi, Germany), and the cells were centrifuged for 45 min (37°C, 4,000 rpm). The supernatant with nonattached planktonic cells was transferred into new wells. The wells with attached cells were washed three times with 500 μl tethering buffer, and the presence of attached cells was verified with phase-contrast microscopy. Fresh TB (300 μl) was subsequently added to the wells, and the plates were incubated at 37°C. The measurements of P*fliC-gfp* activity in attached and planktonic cells were performed with flow cytometry after 60 and 90 min of incubation.

### Proteomic analysis.

To compare the proteomes of WT E. coli Z36 and Δ*motA* strains grown on the surfaces of soft agar plates and in the liquid medium, the cells were grown on the surface of 0.5% TBA for 4 h at 37°C. Flagellar gene expression was confirmed with flow cytometry using P*fliC-gfp* promoter fusion activity as a readout. The cells were then washed off the plates with 2 ml cold phosphate-buffered saline (PBS) (8 g NaCl, 0.2 g KCl, 1.42 g Na_2_HPO_4_, 0.27 g KH_2_PO_4_ per liter), and the final sample size was adjusted to 5 ml with an OD_600_ of 0.6. The cells were then washed twice in 5 ml cold PBS, and the pellets were stored at –20°C. Before the samples were frozen, 50 μl of the suspension was reinoculated into 5 ml of liquid TB and grown at 37°C with shaking to an OD_600_ of 0.6. The cells were then washed twice in 5 ml cold PBS, and the pellets were stored at –20°C. The collected samples were subsequently subjected to proteomic analysis using mass spectrometry and subsequent data analysis as described before (44, 45). Although no reference database for E. coli Z36 is available, using the E. coli CFT073 protein database resulted in full coverage of the analyzed proteome.
