# Supplementary material for: Flagellum-Mediated Mechanosensing and RflP Control Motility State of Pathogenic Escherichia coli
Source: mBio. 2020 Mar 24;11(2):e02269-19. doi: 10.1128/mBio.02269-19 (PMC7157525; doi:10.1128/mBio.02269-19)
Supplement: TABLE S1 [file mBio.02269-19-st001.docx]

**Table S1.** **List of bacterial strains and plasmids used in this study.**

| **Strains** | | **Relevant genotype or phenotype*^a^*** | **Reference or source** | |
| --- | --- | --- | --- | --- |
| U5/41 | | wild-type strain | Leibniz Insitute DSMZ - German Collection of Microorganisms and Cell Cultures (Braunschweig, Germany) | |
| DSM 50902 | | wild-type strain |  |  |
| QST 40139 | | wild-type strain |  |  |
| AMC 198 | | wild-type strain |  |  |
| E611 | | wild-type strain |  |  |
| E2808 | | wild-type strain |  |  |
| EW2129-54 | | wild-type strain |  |  |
| C771 | | wild-type strain |  |  |
| ICB 4004 | | wild-type strain |  |  |
| S13 | | wild-type strain |  |  |
| IHE3035 | | wild-type strain |  |  |
| M185/1-1 | | wild-type strain |  |  |
| B185/29-10 | | wild-type strain |  |  |
| D699(U5/41 Oac-) | | wild-type strain |  |  |
| IHE3043 | | wild-type strain |  |  |
| Z36 | | wild-type strain |  |  |
| T111 | | wild-type strain |  |  |
| PC 0886 | | wild-type strain |  |  |
| CDC 5624-50 | | wild-type strain |  |  |
| *E. coli* MG1655 | | F- lambda- *ilvG*- *rfb*-50 *rph*-1 | (1) | |
| VS-S2-40 | | Z36 Δ*rflP* Km^s^ | This work | |
| VS-S2-99 | | Z36 Δ*motA* Km^s^ | This work | |
| VS-S2-108 | | Z36 Δ*fliC* Km^s^ | This work | |
| VS-S2-118 | | Z36 Δ*fliC* Δ*rflP* Km^s^ | This work | |
| VS-S2-130 | | Z36 Δ*motA* Δ*rflP* Km^s^ | This work | |
| VS-S2-150 | | Z36 *qseC::Km ^r^* | This work | |
| **Plasmids** | | | | |
| pTrc99A | Amp^r^; Expression vector; pBR ori; *trc* promoter, IPTG-inducible | | | (2) |
| pUA66 | Km^r^; Expression vector; SC101 ori, GFPmut2 under control of promoter of interest | | | (3) |
| pKD46 | *repA101*(ts) *oriR101* *bla* P*arab*-(*gam bet exo*) | | | (4) |
| pCP20 | Amp^r^, Cam^r^; *flp* | | | (5) |
| pLeoL18 | Km^r^; P*rflP-gfp* in pUA66 | | | This work |
| pVS1962 | Km^r^; P*flhD-gfp* in pUA66 | | | (6) |
| pVS1963 | Km^r^; P*fliA-gfp* in pUA66 | | | (3) |
| pVS1970 | Km^r^; P*fliC-gfp* in pUA66 | | | (6) |
| pBN5 | Amp^r^; *fliC* in pTrc99A | | | (6) |
| pBN7 | Amp^r^; *motAB* in pTrc99A | | | (6) |
| pLeoL20 | Amp^r^; *rflP*-FLAG in pTrc99A | | | This work |

*^a^*Km^s^, kanamycin sensitive; Km^r^, kanamycin resistant, Amp^r^, ampicillin resistant; Cam^r^, chloramphenicol resistant.

1. Blattner FR, *et al*. 1997. The complete genome sequence of *Escherichia coli* K-12. Science 277:1453–1462.

2. Amann E, Ochs B, Abel K-J. 1988. Tightly regulated *tac* promoter vectors useful for the expression of unfused and fused proteins in *Escherichia coli*. Gene 69:301–315.

3. Zaslaver A, Bren A, Ronen M, Itzkovitz S, Kikoin I, Shavit S, Liebermeister W, Surette MG, Alon U. 2006. A comprehensive library of fluorescent transcriptional reporters for *Escherichia coli*. Nat Methods 3:623–8.

4. Datsenko KA, Wanner BL. 2000. One-step inactivation of chromosomal genes in *Escherichia coli* K-12 using PCR products. Proc Natl Acad Sci U S A 97:6640–6645.

5. Cherepanov PP, Wackernagel W. 1995. Gene disruption in *Escherichia coli*: TcR and KmR cassettes with the option of Flp-catalyzed excision of the antibiotic-resistance determinant. Gene 158:9–14.

6. Ni B, Ghosh B, Paldy FS, Colin R, Heimerl T, Sourjik V. Evolutionary remodeling of bacterial motility checkpoint control. Cell Rep 18:866–877.
