# Supplementary figures and images for: Flagellum-Mediated Mechanosensing and RflP Control Motility State of Pathogenic Escherichia coli
Source: mBio. 2020 Mar 24;11(2):e02269-19. doi: 10.1128/mBio.02269-19 (PMC7157525; doi:10.1128/mBio.02269-19)

**A**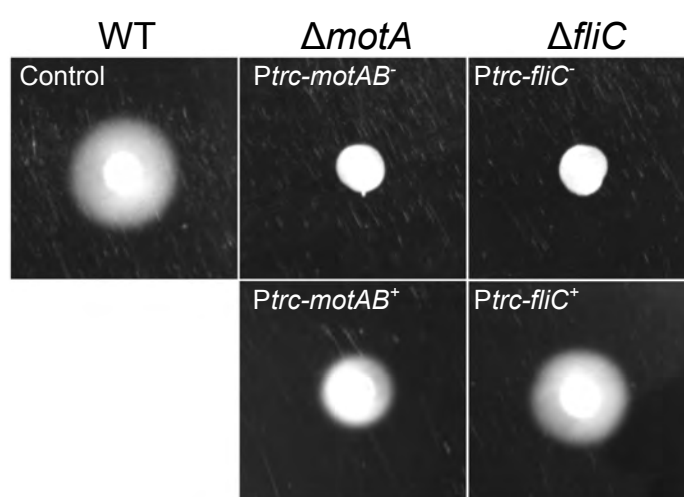**B**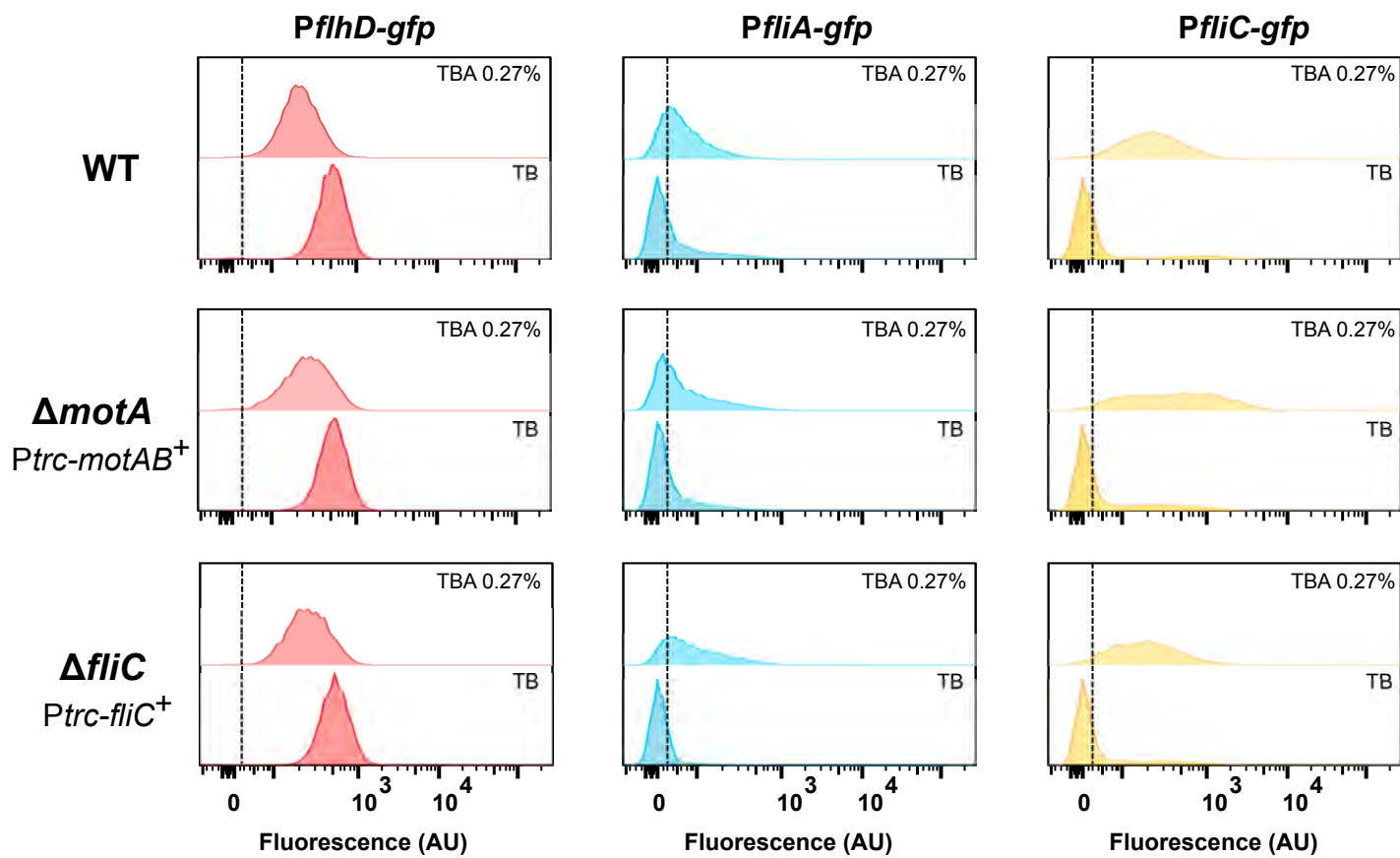

Supplement: FIG S3 [file mBio.02269-19-sf003.pdf]
