# Supplementary material for: Flagellum-Mediated Mechanosensing and RflP Control Motility State of Pathogenic Escherichia coli
Source: mBio. 2020 Mar 24;11(2):e02269-19. doi: 10.1128/mBio.02269-19 (PMC7157525; doi:10.1128/mBio.02269-19)
Supplement: TABLE S2 [file mBio.02269-19-st002.docx]

**Table S2. Proteins with MotA-independent changes in expression between TBA 0.5% and liquid TB as revealed by whole-proteome analysis of *E. coli* Z36.** Shown are only proteins with ≥3-fold change between samples.

| **Protein** | **Function** | **Fold change** | |
| --- | --- | --- | --- |
|  |  | **WT_liq_->WT_surf_** | **∆*motA*_liq_**  **->∆*motA*_surf_** |
| CysA | Sulfate/thiosulfate import ATP-binding protein | -130.13 | -110.11 |
| GadC | Probable glutamate/gamma-aminobutyrate antiporter | -55.27 | -52.50 |
| MetE | 5-methyltetrahydropteroyltriglutamate--homocysteine methyltransferase | -46.73 | -292.50 |
| GadA | Glutamate decarboxylase alpha | -35.40 | -30.62 |
| YbiO | Uncharacterized protein | -26.26 | -247.85 |
| C2453 | Putative polyketide synthase | -21.22 | 3.11 |
| GlsA | Glutaminase | -18.17 | -10.39 |
| YafE | Uncharacterized protein | -14.77 | -8.25 |
| YbgS | Uncharacterized protein | -12.69 | -12.58 |
| GadB | Glutamate decarboxylase beta | -11.83 | -14.67 |
| C3736 | Putative enzyme | -11.60 | -62.44 |
| HdeB | Acid stress chaperone | -10.40 | -9.56 |
| Slp | Outer membrane protein | -10.30 | -11.73 |
| MdtE | Multidrug resistance protein | -10.11 | -10.31 |
| YjbJ | UPF0337 domain protein | -8.78 | -8.51 |
| IlvG | Acetolactate synthase | -8.15 | -12.20 |
| AdhP | Alcohol dehydrogenase | -8.04 | -6.53 |
| YgdI | Uncharacterized lipoprotein | -7.63 | -7.04 |
| OtsB | Trehalose 6-phosphate phosphatase | -7.38 | -6.10 |
| MdtF | Multidrug resistance protein | -7.38 | -6.24 |
| OtsA | Trehalose-6-phosphate synthase | -6.68 | -4.42 |
| DctR | HTH-type transcriptional regulator | -6.53 | -130.97 |
| OsmC | Osmotically inducible protein | -6.39 | -4.72 |
| YicS | Uncharacterized protein | -6.37 | -3.04 |
| C1843 | Glyceraldehyde-3-phosphate dehydrogenase | -6.34 | -4.59 |
| Tam | Trans-aconitate 2-methyltransferase | -6.31 | -5.25 |
| YghA | Hypothetical oxidoreductase | -6.27 | -4.62 |
| CysP | Thiosulfate-binding protein | -6.26 | -5.15 |
| MetA | Homoserine O-succinyltransferase | -5.98 | -6.95 |
| HdeA | Acid stress chaperone | -5.63 | -6.87 |
| PhnB | Putative polyketide synthase | -5.50 | -15.08 |
| Sra | Stationary-phase-induced ribosome-associated protein | -5.30 | -4.85 |
| C4774 | Uncharacterized protein | -5.28 | -5.59 |
| SufD | FeS cluster assembly protein | -5.20 | -11.18 |
| CsgG | Curli production assembly/transport component | -5.18 | -8.79 |
| FbaB | Fructose-bisphosphate aldolase class 1 | -5.03 | -4.79 |
| AidB | Putative acyl-CoA dehydrogenase | -5.03 | -5.30 |
| OsmY | Osmotically-inducible protein | -4.96 | -3.88 |
| YgaM | Uncharacterized protein | -4.96 | -4.28 |
| FhlA | Formate hydrogenlyase transcriptional activator | -4.81 | -3.23 |
| YegP | UPF0339 protein | -4.81 | -3.23 |
| EcnB | Entericidin B | -4.64 | -4.22 |
| YbeM | Putative amidase | -4.58 | -4.59 |
| MsrP | Protein-methionine-sulfoxide reductase catalytic subunit | -4.57 | -7.47 |
| YjcH | Uncharacterized protein | -4.54 | -5.28 |
| AldB | Aldehyde dehydrogenase | -4.53 | -4.72 |
| YcfS | Uncharacterized protein | -4.49 | -5.89 |
| CysJ | Sulfite reductase [NADPH] flavoprotein alpha-component | -4.43 | -3.75 |
| GabT | 4-aminobutyrate aminotransferase | -3.97 | -4.68 |
| YcaC | Putative hydrolase | -3.88 | -3.84 |
| ActP | Cation/acetate symporter | -3.87 | -3.83 |
| ProV | Glycine betaine/L-proline transport ATP-binding protein | -3.87 | -9.19 |
| PhnO | Aminoalkylphosphonate N-acetyltransferase | -3.83 | -3.72 |
| TktB | Transketolase | -3.82 | -3.71 |
| YahK | Hypothetical zinc-type alcohol dehydrogenase-like protein | -3.78 | -3.11 |
| PtrB | Protease II | -3.73 | -3.52 |
| ChuY | Uncharacterized protein | -3.66 | -3.39 |
| FimF | Fimbrial protein | -3.63 | -3.24 |
| Prr | Gamma-aminobutyraldehyde dehydrogenase | -3.59 | -4.04 |
| CsiD | CsiD family protein | -3.54 | -4.56 |
| YebV | Uncharacterized protein | -3.53 | -4.56 |
| CysN | Sulfate adenylyltransferase subunit | -3.52 | -9.77 |
| YgaU | Uncharacterized protein | -3.48 | -3.03 |
| GabD | Succinate-semialdehyde dehydrogenase (NADP+) | -3.47 | -3.81 |
| FimG | Fimbrial protein | -3.44 | -4.67 |
| YehZ | Uncharacterized protein | -3.42 | -3.32 |
| YgaF | L-2-hydroxyglutarate oxidase | -3.36 | -4.07 |
| CysH | Phosphoadenosine phosphosulfate reductase | -3.31 | -5.99 |
| ProX | Glycine betaine-binding periplasmic protein | -3.31 | -3.56 |
| PatA | Putrescine aminotransferase | -3.16 | -5.47 |
| YjgR | Uncharacterized protein | -3.16 | -3.08 |
| YdfI | Hypothetical oxidoreductase | -3.13 | -3.84 |
| TdcD | Propionate kinase | 3.00 | 4.49 |
| NanM2 | N-acetylneuraminate epimerase 2 | 3.08 | 4.64 |
| YjiY | Uncharacterized protein | 3.31 | 5.48 |
| GlpC | Anaerobic glycerol-3-phosphate dehydrogenase subunit | 3.78 | 4.93 |
| DsdA | D-serine dehydratase | 3.94 | 3.75 |
| YcgZ | Uncharacterized protein | 4.29 | 3.37 |
| YecR | Uncharacterized protein | 4.30 | 12.55 |
| FdnG | Formate dehydrogenase, nitrate-inducible, major subunit | 5.99 | 9.24 |
| PhoH | Phosphate starvation inducible protein | 6.57 | 5.65 |
| DsdX | D-serine transporter | 9.21 | 11.63 |
| IbpB | Small heat shock protein | 9.26 | 8.12 |
| CadA | Lysine decarboxylase | 12.53 | 20.32 |
| YlaC | Uncharacterized protein | 23.49 | 3.08 |
| YhaM | UPF0597 protein | 25.80 | 10.83 |
| YciI | Uncharacterized protein | 216.47 | -4.02 |
| Hda | DnaA regulatory inactivator | 273.69 | 184.14 |
